# The Germ Fraction Inhibits Iron Bioavailability of Maize: Identification of an Approach to Enhance Maize Nutritional Quality via Processing and Breeding

**DOI:** 10.3390/nu11040833

**Published:** 2019-04-12

**Authors:** Raymond Glahn, Elad Tako, Michael A. Gore

**Affiliations:** 1 United States Department of Agriculture, Agricultural Research Service, Robert Holley Center for Agriculture and Health, 538 Tower Road, Ithaca, NY 14853, USA; elad.tako@ars.usda.gov; 2 Plant Breeding and Genetics Section, School of Integrative Plant Science, Cornell University, Ithaca, NY 14853, USA; mag87@cornell.edu

**Keywords:** maize, iron, bioavailability, germ, Caco-2, in vitro digestion, bioassay, biofortification

## Abstract

Improving the nutritional quality of Fe in maize (*Zea mays*) represents a biofortification strategy to alleviate iron deficiency anemia. Therefore, the present study measured iron content and bioavailability via an established bioassay to characterize Fe quality in parts of the maize kernel. Comparisons of six different varieties of maize demonstrated that the germ fraction is a strong inhibitory component of Fe bioavailability. The germ fraction can contain 27–54% of the total kernel Fe, which is poorly available. In the absence of the germ, Fe in the non-germ components can be highly bioavailable. More specifically, increasing Fe concentration in the non-germ fraction resulted in more bioavailable Fe. Comparison of wet-milled fractions of a commercial maize variety and degerminated corn meal products also demonstrated the inhibitory effect of the germ fraction on Fe bioavailability. When compared to beans (*Phaseolus vulgaris*) containing approximately five times the concentration of Fe, degerminated maize provided more absorbable Fe, indicating substantially higher fractional bioavailability. Overall, the results indicate that degerminated maize may be a better source of Fe than whole maize and some other crops. Increased non-germ Fe density with a weaker inhibitory effect of the germ fraction are desirable qualities to identify and breed for in maize.

## 1. Introduction

Biofortification was a term officially coined in 2001 and became a formal strategy for nutritional enhancement of staple crops around 2003. It was at this time that the organization known as HarvestPlus was formed and headquartered at the International Food Policy Research Institute in Washington, DC. Biofortification has its early roots in the United States Department of Agriculture (USDA) research program at centers such as the Plant, Soil and Nutrition Lab in Ithaca, NY (now known as the Robert Holley Center for Agriculture and Health), and in various CGIAR (Consultative Group for International Agricultural Research) Centers worldwide (harvestplus.org). The biofortification research objective is simply to improve human health via enhancement of the nutritional content in staple food crops of the micronutrients vitamin A, zinc (Zn), and iron (Fe). These three micronutrients represent the majority of the “hidden hunger” affecting approximately two billion people worldwide.

Maize (*Zea mays*) is the cereal crop with the highest production worldwide, consistently ranking third or higher as a staple food behind wheat and rice [1]. Biofortification of maize therefore has the potential to have a major impact on human health. Maize biofortifcation efforts have focused primarily on increasing Vitamin A content, with significant advances evidenced by release of enhanced varieties in Nigeria and Zambia [2]. In contrast to Vitamin A, iron biofortification of maize has been explored with limited success using in vitro (Caco-2 cell bioassay) and in vivo (poultry) models for Fe bioavailability [3]. In vitro studies of tropical maize varieties from Nigeria documented relatively small varietal differences in Fe and Zn content, and in Fe bioavailability [4,5,6]. Although some varieties were identified as promising, they were not pursued further. Additional in vitro studies on lines of superior hybrids from the International Maize and Wheat Improvement Center (CYMMYT) were also conducted [7]. Overall, this study of CYMMT lines was encouraging as it demonstrated that breeding for Fe bioavailability and Fe concentration were distinct and largely unrelated traits; however, the environmental effects were large for both traits, and no follow-up studies were conducted. 

Subsequent studies using the combination of Caco-2 cell bioassay and the poultry model developed maize varieties with high Fe bioavailability [8]. This research utilized the Caco-2 cell bioassay to guide identification of quantitative trait loci (QTL) in an established recombinant inbred population, essentially identifying genetic regions important to Fe concentration and Fe bioavailability. Animal feeding trials confirmed the in vitro approach to marker-assisted breeding, demonstrating that Fe bioavailability of maize could be highly enhanced, and that such varieties can be produced in large quantity [9]. However, in a subsequent retraction note [10], the authors point out that the in vitro and in vivo results of that work were indeed valid, and that these lines clearly demonstrated enhanced Fe bioavailability. Retraction of the paper was because the genotypes were not isogenic and homozygous for the genetic regions as originally described. Due to this research setback, regeneration of the enhanced lines has not yet occurred.

It should be noted that in the previously mentioned study, the enhanced lines were equal in Fe content to the controls [9]. Furthermore, the scientists were unable to find specific compounds, such as polyphenolics or lower phytate, in one variety that could explain the difference in bioavailability. Thus, there appears to be some other factor(s) that influences the Fe bioavailability from maize. 

The present study therefore seeks to address gaps in knowledge related to Fe concentration and bioavailability in the maize kernel, as such information could shed light on a strategy for Fe biofortification of maize. Two key observations contributed to the design of the present study. First, it is a common observation that diversity populations of maize exhibit significant differences in the amount of floury and horny endosperm present in the seed. Second, a thorough review of the literature also shows that very little has been published on the concentration and relative amounts of Fe in the various parts of the maize kernel; and, there are no reports of Fe bioavailability from the individual components of the maize kernel. Therefore, the objectives of the present study were to determine the relative concentration and bioavailability of Fe in the germ and non-germ fraction of the maize kernel, and to investigate the Fe concentration and bioavailability of fractions generated during commercial wet-milling of maize.

## 2. Materials and Methods 

### 2.1. Chemicals, Enzymes, and Hormones

All chemicals, enzymes, and hormones were purchased from Sigma Chemical Co. (St. Louis, MO, USA) unless stated otherwise.

### 2.2. Sample Source and Preparation

**Figure 1:**
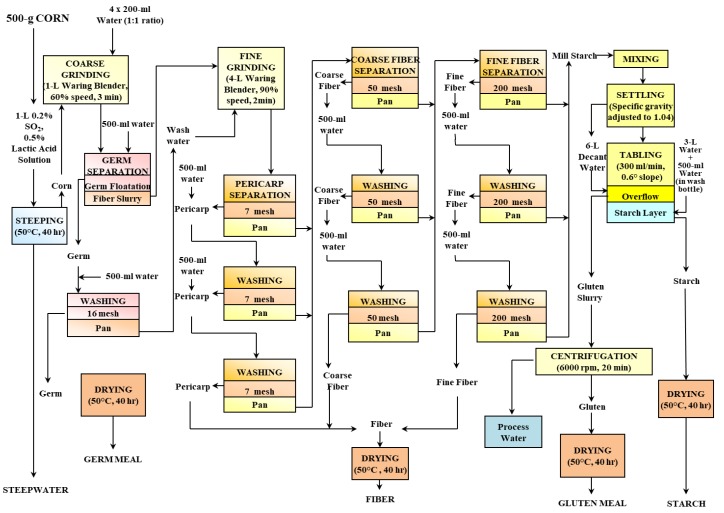
Diagram of the wet-milling process used in the present study. Image courtesy of Mr. Steven Fox, Center for Crops Utilization Research, Iowa State University, Ames, IA.

A 500 g sample of a commercial maize variety (Pioneer 3245, Iowa State University, Ames, IA, USA) was subjected to a wet-milling process at the Center for Crops Utilization Research, Iowa State University, Ames, Iowa. The diagram presented in Figure 1 illustrates the wet-milling procedure and where fractions were extracted. 

**Figure 2:**
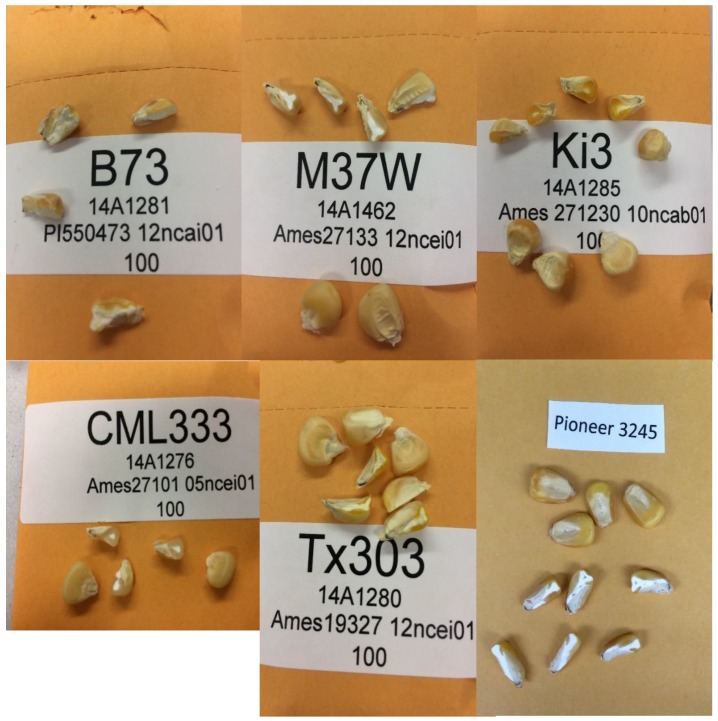
Images of maize kernels analyzed in the present study. Note that some kernels are split longitudinally to expose visual differences between varieties.

In 2014, a set of five maize inbred lines (B73, CML333, Ki3, M37W, Tx303) ranging from dent to flint kernel types was evaluated at Cornell University’s Musgrave Research Farm in Aurora, NY. These five inbred lines were selected to span a range of whole-kernel Fe concentrations (Gore, unpublished data). Conventional maize cultivation practices for the northeastern United States were used. Self-pollinated ears were hand-harvested at physiological maturity and dried with forced air to 10–15% kernel moisture. The dried ears of each plot were manually shelled and bulked to form a representative sample from which 100 kernels were randomly selected. Whole kernels of the Pioneer 3245 were also obtained from the Iowa facility. For all six varieties mentioned above, a scalpel was used to remove the tip cap from each kernel, followed by a center longitudinal excision, followed by excision of the germ tissue. This was done for each kernel in the sample. The germ and the non-germ fractions (which included the aleurone and pericarp) were weighed for subsequent calculations related to content. As shown in Figure 2, the samples exhibited diversity in kernel size, floury endosperm, and horny endosperm composition. 

Maize samples purchased in a local supermarket included the following: “Organic Cornmeal”, which is an unfortified ground whole kernel maize flour (19 µg Fe/g); a fortified whole kernel maize flour (65 µg Fe/g); and a degerminated fortified maize flour (44 µg Fe/g). According to the package labels, these products were fortified with “reduced Fe”.

Unfortified whole grain wheat flour (37 µg Fe/g) and an unfortified 80% extraction wheat flour (9 µg Fe/g) were also purchased at a local supermarket.

For comparison of Fe bioavailability, two commercial white bean samples and one red mottled bean were included in the study. The white beans were Merlin Navy beans and the red mottled variety were PR0737-1. The beans samples were grown in research plots in Montcalm, Michigan in the summer of 2015. The dry weight Fe concentrations of the beans were 76, 82, and 89 µg Fe/g, for the white bean 1, white bean 2, and red mottled bean, respectively.

Due to the small sample sizes available for six varieties of maize, all maize samples were not cooked in this study. The bean samples were cooked by autoclave for 15 min in a 3:1 volume of water:bean, then freeze-dried and ground into powder with a common coffee grinder.

### 2.3. Mineral Analysis

Dried, ground food samples (0.5 g) were treated with 3.0 mL of a 60:40 HNO_3_ and HClO_4_ mixture in a Pyrex glass tube and left overnight to destroy organic matter. The mixture was then heated to 120 °C for two hours, and 0.25 mL of 40 µg/g Yttrium was added as an internal standard to compensate for any drift during the subsequent inductively coupled plasma atomic emission spectrometer (ICP-AES) analysis. The model of the ICP used was a Thermo iCAP 6500 series (Thermo Jarrell Ash Corp., Franklin, MA, USA). The temperature of the heating block was then raised to 145 °C for 2 h. If necessary, more nitric acid (1–2 mL) was added to destroy the brownish color of the organic matter. Then, the temperature of the heating block raised to 190 °C for ten minutes and turned off. The cooled samples in the tubes were then diluted to 20 mL, vortexed, and transferred onto auto sampler tubes to be analyzed via ICP-AES. 

### 2.4. In Vitro Digestion

The in vitro digestion protocol was conducted as per an established, highly validated, in vitro digestion model [11,12]. Briefly, exactly 1 g of each sample was used for each sample digestion. To initiate the gastric phase of digestion, 10 mL of fresh saline solution (0.9% sodium chloride) was added to each sample and mixed. The pH was then adjusted to 2.0 with 1.0mol/L HCl, and 0.5 mL of the pepsin solution (containing 1 g pepsin per 50 mL; certified >250 U per mg protein; Sigma #P7000, St. Louis, MO, USA) was added to each mixture. The mixtures were under gastric digestion for 1 h at 37 °C on a rocking platform (model RP-50, Laboratory Instrument, Rockville, MD, USA) located in an incubator. After 1 h of gastric digestion, the pH of the sample mixture was raised to 5.5–6.0 with 1.0 mol/L of NaHCO_3_ solution. Then, 2.5 mL of the pancreatin–bile extract solution was added to each mixture. The pancreatin–bile extract solution contained 0.35 g pancreatin (Sigma #P1750, St. Louis, MO, USA) and 2.1 g bile extract (Sigma #B8631, St. Louis, MO, USA) in a total volume of 245 mL. The pH of the mixture was then adjusted to approximately 7.0, and the final volume of each mixture was adjusted to 15.0 mL by weight using a salt solution of 140 mmol/L of NaCl and 5.0 mmol/L of KCl at pH 6.7. At this point, the mixture was referred to as a “digest”. The samples were then incubated for an additional two hours at 37 °C, at which point the digests were centrifuged, and supernatants and pellet fractions collected and transferred to tubes for analysis. Three independent replications of the in vitro digestion procedure were carried out for all of the food samples. For some samples, as noted in the specific results section, Fe bioavailability was assessed in both the presence and absence of ascorbic acid (AA). The AA was added to the digests at the start of the gastric digestion phase at a concentration of 10 µmol/L. This treatment has been shown to expose some additional differences between samples and thus provides further information on the matrix of the digest.

### 2.5. Statistical Analysis 

Data were analyzed using the software package GraphPad Prism 8 (GraphPad Software, San Diego, CA, USA). Data were analyzed using analysis of variance incorporating normalization of variance, if needed, and Tukey’s post test to determine significant differences (*p* < 0.05) between groups. Unless noted otherwise, values are expressed as mean ± standard deviation (SD); *n* = 3 independent replications.

## 3. Results

### 3.1. Maize Fe Concentration and Fe Bioavailability

**Figure 3:**
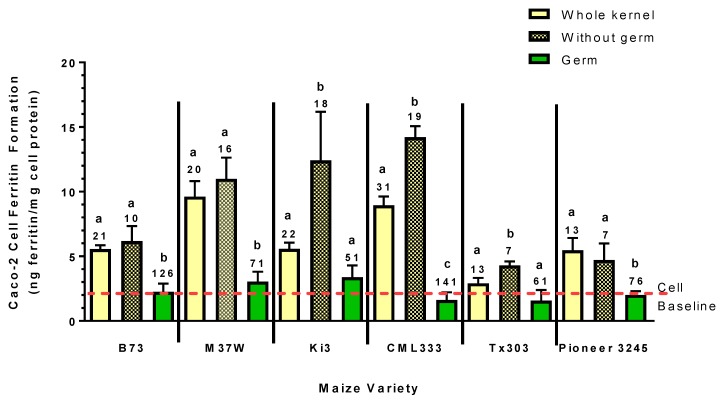
Iron bioavailability from manually dissected maize samples as measured via Caco-2 cell bioassay (cell ferritin formation equals Fe uptake). Numbers on top of the bar values represent Fe concentrations (µg/g) of samples. Bar values within variety with no letter in common are significantly different (*p* < 0.05). Bar values are mean ± SD, *n* = 3. Dashed red line indicates Caco-2 cell baseline ferritin level when exposed only to the in vitro digest solutions without food samples.

Iron concentration in the maize samples ranged from 12.5 to 30.8 µg/g in the whole kernels (Table 1). The germ fractions contained the highest density of Fe, ranging from 51.0 to 141.3 µg/g. Iron concentration in the non-germ component ranged from 7.4 to 18.9 µg/g. It is notable that the three varieties, M37W, Ki3, and CML333, demonstrating the highest Fe bioavailability also had the highest non-germ Fe concentrations: 15.9–18.9 µg/g (Figure 3). The other three varieties exhibited non-germ Fe concentrations of 7.4–10.4 µg/g and thus exhibited lower Fe bioavailability.

The manual dissection of the germ fraction from the maize demonstrated that the germ has the highest density of Fe in the seed, ranging from 51 to 141 µg/g, and accounting for 27–54% of the total Fe (Table 1). The non-germ portion of the kernels from each variety was of lesser Fe density, ranging from 7 to 19 µg/g, and accounting for 46–73% of the total Fe in the seed.

Iron bioavailability from the germ fractions was low for all varieties despite having high Fe concentrations (Figure 3). Despite being significantly lower in Fe content, the non-germ fractions exhibited similar or greater Fe bioavailability than the whole kernel samples; and except for two varieties, M37W and Tx303, the non-germ fractions were higher than the germ fractions. 

### 3.2. Kernel Size

Kernel size was substantially different among the varieties but was not measured in this study; however, the average germ fraction by weight was 9.4%, ranging from 7.8 to 11.7% across the six samples. The commercial line, Pioneer 3245, was clearly the largest in terms of kernel size, with a substantial portion of the kernel comprised of floury endosperm. In contrast, the CML333 and Ki3 samples were smaller kernels, with lesser amounts of floury endosperm (Figure 2).

### 3.3. Maize Phosphorous Concentration

Due to limited amounts of material, kernel phosphorous levels were used as an indirect estimate of phytic acid levels. The results clearly show that P was more concentrated in the germ fraction of all varieties. Phosphorous density was highest in the germ fraction ranging from 14 to 23 mg/g, and accounting for 55–65% of the total seed P (Table 1). The non-germ fraction of the kernel was less dense in P, ranging from 0.9 to 1.7 mg/g, and accounting for 35–45% of the total seed P.

### 3.4. Wet-Milled Fractions of Maize

**Figure 4:**
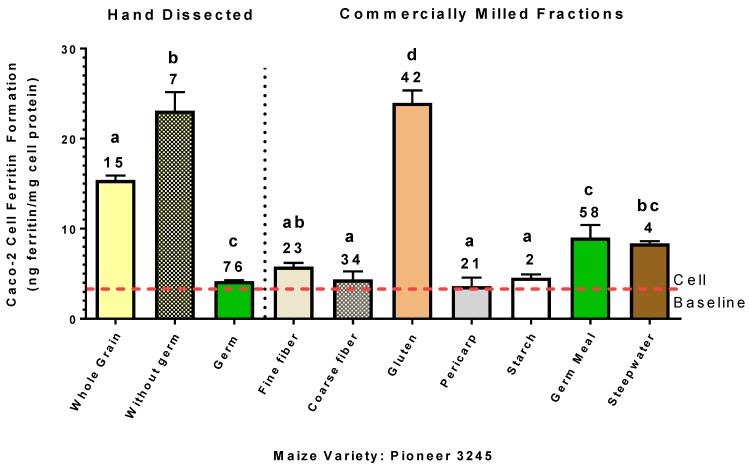
Iron bioavailability from the Pioneer 3245 maize samples that were manually dissected versus wet-milled fractions from a 500 g sample. Caco-2 cell ferritin values represent relative Fe bioavailability as cell ferritin formation is proportional to Fe uptake. Numbers on top of bar values represent Fe concentrations (µg/g) of samples. Bar values within manually dissected or commercial fractions with no letter in common are significantly different (*p* < 0.05). Bar values are mean ± SD, *n* = 3. Dashed red line indicates Caco-2 cell baseline ferritin level when exposed only to the in vitro digest solutions without food samples.

Commercial milling of the Pioneer 3245 variety resulted in the gluten fraction containing the highest Fe bioavailability (Figure 4). The germ fraction was the highest in Fe concentration but significantly lower in bioavailable Fe relative to the gluten fraction. Compared to the hand dissected germ fraction of the Pioneer 3245 variety, it appears that the milled germ fraction was less pure relative to the dissected germ fraction. The Fe in the fiber fractions and the pericarp were poorly available as indicated by ferritin values similar to the cell baseline, which indicates no net increase in Fe uptake as a result of exposure to the in vitro digest. The starch fraction contained only trace levels of Fe (2 µg/g). The steep liquor contained only 4 µg/g and yet exhibited a noticeable increase in ferritin above the cell baseline, indicating that this small amount of Fe could be highly available.

Iron and P analysis of the wet-milled Pioneer 3245 fractions showed that 29.3% of the total Fe was in the gluten fraction, followed by the germ fraction with 16.8% (Table 2). The concentration of Fe in the germ fraction (58.2 µg/g) was higher than that of the gluten fraction (42 µg/g); however, the P concentration in the gluten fraction was substantially less than that of the germ fraction, which contained seven times more P. This suggests that the phytate:Fe molar ratio was significantly less in the gluten fraction and thus may explain the relatively high Fe bioavailability of the gluten fraction.

### 3.5. Comparison of Supermarket Samples vs. Degerminated Maize

**Figure 5:**
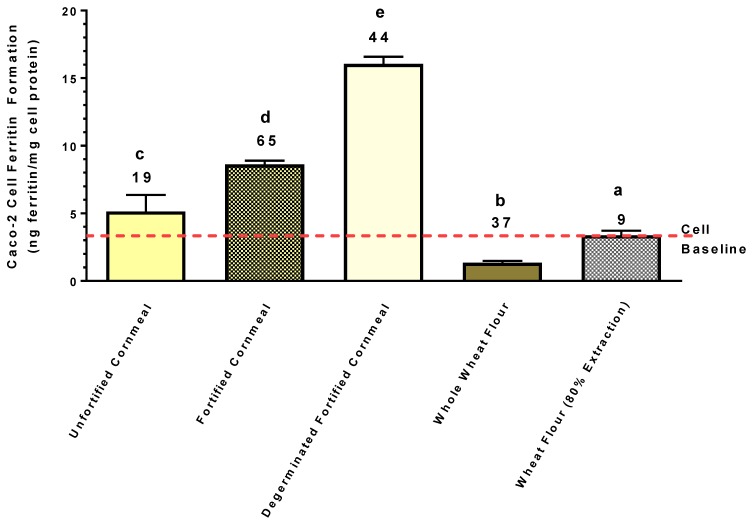
Iron bioavailability from the cornmeal and wheat flours purchased at a local supermarket. Caco-2 cell ferritin values represent relative Fe bioavailability as cell ferritin formation is proportional to Fe uptake. Numbers on top of bar values represent Fe concentrations (µg/g) of samples. Bar values with no letter in common are significantly different (*p* < 0.05). Bar values are mean ± SD, *n* = 3. Dashed red line indicates Caco-2 cell baseline ferritin level when exposed only to the in vitro digest solutions without food samples. A bar value below the baseline indicates strong inhibitory factors that negate the bioavailability of ultra-low background Fe present in the digest solutions.

Of the cornmeal products purchased in the local supermarket, two were fortified with Fe (Figure 5). Comparison of these samples indicated that the absence of the germ fraction improved Fe bioavailability from the fortified cornmeal. The unfortified whole wheat sample was lower in bioavailable Fe relative to the maize samples, and also lower relative to the 80% extracted wheat flour.

**Figure 6:**
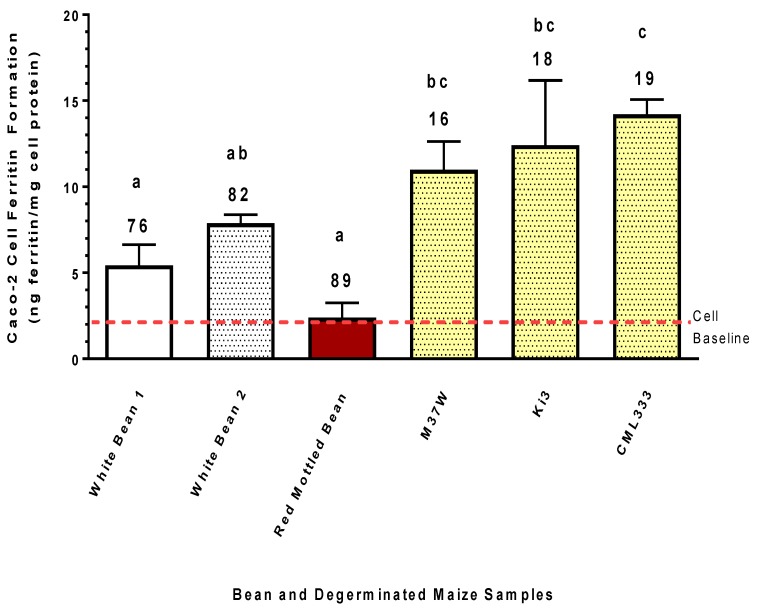
Iron bioavailability from white bean, red bean, and degerminated maize samples. Caco-2 cell ferritin values represent relative Fe bioavailability as cell ferritin formation is proportional to Fe uptake. Numbers on top of bar values represent Fe concentrations (µg/g) of samples. Bar values with no letter in common are significantly different (*p* < 0.05). Bar values are mean ± SD, *n* = 3. Dashed red line indicates Caco-2 cell baseline ferritin level when exposed only to the in vitro digest solutions without food samples. A bar value below the baseline indicates strong inhibitory factors that negate the bioavailability of ultra-low background Fe present in the digest solutions.

A direct comparison of Fe bioavailability of white beans, red-mottled beans, and degerminated maize samples is shown in Figure 6. These samples were compared on an equal dry weight basis (0.5 g sample per replicate) in the same run of the bioassay. Relative to the beans, more Fe was taken up by the Caco-2 cells from the degerminated maize samples versus the beans. This occurred despite the fact that the bean samples contained approximately 80% more Fe than the maize. This observation indicated greater fractional bioavailability of the Fe in the maize samples relative to the beans.

## 4. Discussion

The present study clearly shows that the Fe in the germ fraction of maize is very low in bioavailability (Figure 3). This was evident by the observation that the Caco-2 cell ferritin formation (i.e., the measure of Fe uptake) from the germ fraction of all six varieties was essentially equal to the baseline ferritin of the cells, despite having Fe concentration of 51–126 µg/g. In addition, the germ fraction appears to inhibit Fe bioavailability from the rest of the kernel. This effect was also evident in all six of the maize varieties analyzed, as Caco-2 cell ferritin formation from the degerminated kernel was either equal to or greater than that of the whole kernel, despite a decrease in Fe content by approximately 20–50% (Figure 3).

Iron bioavailability and Fe content analyses of the commercially wet-milled fractions of the Pioneer 3245 variety also demonstrated that the Fe of the germ fraction was poorly available (Figure 4; Table 2). However, in this experiment, the ferritin formation value for the “germ meal” fraction indicated that more bioavailable Fe was present. Given that the germ meal fraction was lower in Fe concentration relative to the hand-dissected fraction (58 vs. 76 µg/g, respectively), it would reasonably suggest that the wet-milled germ fraction was not as pure in germ composition as the manually excised germ tissue. Alternatively, the wet-milling process may alter the Fe bioavailability, perhaps through dilution in the wet-milling or loss of phytate via endogenous phytase activity during processing. Interestingly, the “gluten” fraction demonstrated the highest Fe bioavailability with an Fe concentration of 42 µg/g. The gluten fraction was also significantly lower in P concentration relative to the germ meal fraction, suggesting the phytate:Fe molar ratio was significantly less in the gluten fraction and thus contributing to the higher Fe bioavailability. Not surprisingly, the fiber fractions and the pericarp were of low Fe bioavailability despite Fe concentrations of 23–34 µg/g. In contrast, the “steepwater” fraction with an Fe concentration of only 4 µg/g yielded a ferritin formation value equal to or greater than that of the pericarp and fiber fractions. Polyphenol analysis of the steepwater was inconclusive as no polyphenols known to promote or inhibit Fe uptake were detected. 

Until the present study, very little information was available on the distribution of Fe in maize, and there appears to be no reports of the relative Fe bioavailability between maize fractions. The present study clearly shows in this small set of samples that the germ tissue Fe content can vary substantially, ranging in this study from 27 to 54% of the total kernel Fe; thus, the non-germ fraction can have between 46 to 73% of the total kernel Fe. This relative Fe content suggests that breeding for higher non-germ Fe content may be possible; however, the question remains as to whether or not such an increase could overcome the inhibitory effect of the germ fraction. Such concern is particularly relevant to populations that consume maize as a major dietary staple, where Fe deficiency is prevalent. A broader set of diverse maize samples should clearly be evaluated to address the feasibility of these potential breeding objectives. 

The present study clearly indicates that degermed maize and degermed maize products found in the marketplace can provide more absorbable Fe than crops such as beans or wheat, even though these crops have substantially more Fe (Figure 5 and Figure 6). It is important to keep in mind that this Caco-2 bioassay gives a relative measure of the amount of Fe absorbed from the amount of sample provided. Therefore, the results indicate that relative to the bean and wheat samples used in the present study, the degermed maize was a better source of Fe, exhibiting a higher fractional Fe bioavailability and delivering more Fe to the enterocytes. Such observations suggest that Fe biofortification of maize could have a more profound impact relative to beans and wheat on improving Fe status of populations prone to Fe deficiency.

Milling of maize to remove the germ fraction and thus potentially promote Fe absorption in at risk populations may not be a viable or affordable option on a large scale. Indeed, many regions of Africa and Latin America are challenged by having enough maize that is uncontaminated by mycotoxins [13]. However, it is likely that in some regions or for some food applications such as infant complementary foods, a degerminated maize product could be produced, or is already being produced, that would be acceptable to consumers. For that reason alone, further research on the application of degermed maize to improve Fe nutrition is warranted. Animal and or human studies should be conducted to confirm this in vitro observation. If this effect is demonstrated in vivo, then it is clearly an intervention that could be deployed immediately to improve Fe nutrition of maize-based foods. 

Iron biofortification research has primarily focused on crops that have been shown to be relatively high in iron and demonstrate a genetic diversity for Fe content [14]. For example, common beans have been shown to have diversity in Fe content, ranging from 50 to 100 µg/g [15,16], and pearl millet has shown a range of 30–80 µg/g [17]. Improvement in Fe status following prolonged consumption has been demonstrated in humans and in an animal model when the Fe concentration in the common bean or pearl millet has been compared at these extreme levels [18,19,20]. There is also evidence in a poultry model, where a lesser difference in the Fe concentration in beans, 50 vs. 71 µg/g, can have significant benefit [21]. It should be noted that the key assumptions of this approach is that increased Fe concentration and content can deliver additional absorbable Fe within the overall diet, and that such increases in Fe are primarily genetic and can be stable traits. 

In contrast to beans, maize has not been extensively pursued for Fe biofortification, possibly because the Fe concentration is approximately 60% lower, and because there is a general perception that Fe bioavailability in maize is very low [14,22]. The low Fe bioavailability is believed to be primarily due to phytic acid, as polyphenols known to influence Fe bioavailability have not been identified as a factor in yellow or white maize varieties (R.P. Glahn, unpublished observations) [23]. Colored varieties of maize certainly have substantial amounts of polyphenols, but these have not been evaluated for Fe bioavailability or polyphenol profiles. Mutant varieties of maize with low phytic acid levels have been developed, but appear to have limited application, primarily due to reduced seedling viability [24]. A transgenic strategy to enhance the nutritional quality of Fe in low phytate maize, the overexpression of soybean ferritin in the endosperm, has been explored and appears to enhance the amount of absorbable Fe [25]. However, a search of the literature finds no additional research on this approach, nor on any large-scale production of low-phytate maize, suggesting that for maize, this approach has been abandoned.

To date, Fe biofortification of staple food crops has focused primarily on crops such as beans and pearl millet, as these crops demonstrate a diversity in range with relatively high Fe content. The assumption in focusing on these crops is that more Fe content should result in delivering more Fe for absorption, and that the Fe content is a genetically tractable trait. In addition, such assumptions require that sufficient bioavailability accompanies the added Fe. The results of the present study clearly suggest an alternative approach to Fe biofortification. More precisely, this study suggests that focusing on identifying the foods and food matrices that result in more Fe may be a more effective and efficient approach. Even though this study presents only in vitro data, it should be noted that the Caco-2 bioassay correctly predicted the beneficial effects of high Fe carioca beans and high Fe pearl millet in human efficacy trials [12]. The Caco-2 cell ferritin values for those crops were much lower on a relative scale than those of the degermed maize samples analyzed in this study. Granted, there are additional nutritional reasons for consuming crops such as beans; however, for many populations, food choices are limited, and to alleviate iron deficiency anemia, all strategies should be considered to improve the nutritional quality of Fe in the food system.

The present study is also evidence that this model is capable of identifying new factors and obstacles to improving Fe quality in staple foods. For example, this model has shown that the cotyledon cell wall of beans represents a significant barrier to the bioaccessibility of Fe in the bean cotyledon [26]. Moreover, it has shown that such obstacles can be overcome by processing or possibly by breeding for faster cooking, a tractable trait that is likely related to cell wall structure and linked to improved Fe bioavailability [27]. This model has also identified seed coat polyphenols, and thus color classes of beans that may be better sources of Fe nutrition [28,29]. In vitro observations should always be viewed with a healthy dose of skepticism; however, as stated previously, this model has correctly predicted numerous in vivo results of Fe biofortified crops. Most recently, this model predicted key interactions between carioca beans and the “food basket” of a common Brazilian diet [30]. Therefore, the in vitro observations from the application of this in vitro approach are now highly accepted as a tool to assess specific foods and diets and to guide plant breeding for enhanced Fe quality.

In summary, by identifying the germ fraction of maize as a significant inhibitor of Fe bioavailability from this crop, and proposing the strategy of increasing the non-germ, mostly endosperm Fe content, an additional breeding strategy is thus exposed to enhance the nutritional quality of maize. It should also be noted that the present study focused only on removing the germ fraction and noted the enhancing effects of removal of the germ fraction. It may be possible to use this approach to identify a germ fraction trait that may be less inhibitory, and thus complement the strategy of increasing Fe content in the non-germ fraction. It is also highly likely that such varieties may already be available in commercial production, and simply need to be identified. Given the broad consumption of maize and the high incidence of Fe deficiency anemia in resource-poor populations, a successful application of the above could have profound effect on human health.

## Figures and Tables

**Table 1:** Iron and phosphorous ^1^ concentration in the germ and non-germ (endosperm + pericarp) fractions of maize varieties. Percent values represent percent of total in whole kernel sample.

Maize Variety	Whole Seed Fe (µg/g)	Germ Fe (µg/g)	Germ Fe %	Non Germ Fe (µg/g)	Non Germ Fe %	Whole Seed P (mg/g)	Germ P (mg/g)	Germ P %	Non Germ P (mg/g)	Non Germ P %
B73	20.6	126.4	54.1	10.4	45.9	2.96	20.8	61.8	1.24	38.2
M37W	20.2	71.0	27.5	15.9	72.5	3.06	22.2	56.7	1.44	43.3
Ki3	21.8	51.0	27.4	17.9	72.6	3.43	16.2	55.4	1.73	44.6
CML333	30.8	141.3	44.7	18.9	55.3	3.38	22.5	64.8	1.32	35.2
Tx303	12.5	61.4	46.3	7.4	53.7	3.16	20.8	62.1	1.33	37.9
Pioneer 3245	13.4	75.8	49.2	7.5	50.8	2.04	14.4	61.4	0.87	38.6

^1^ Phosphorous was used as an indirect estimate of phytic acid content as insufficient material was available to directly measure phytic acid.

**Table 2:** Iron and phosphorous concentration and amounts in the various fractions from the wet-milling process of the Pioneer 3245 variety ^1^.

Fraction	Dry Weight (g)	Percent of Total Solids Recovered	Fe Concentration (µg/g)	Percent of Total Fe	P Concentration (µg/g)	Percent of Total P
Fine Fiber	42.8	9.3	22.6	12.6	686	2.7
Coarse Fiber	27.5	6.0	33.7	12.1	698	1.8
Gluten	53.6	11.6	42.0	29.3	2060	10.2
Pericarp	15.9	3.4	20.9	4.3	234	0.3
Starch	255.2	55.4	2.3	7.6	271	6.4
Germ	22.1	4.8	58.2	16.8	14401	29.5
Steep Liquor	---	---	4.2 *	---	938 **	---

^1^ A 500 g sample of maize was milled. Recovered total solids (dry weight) were 461 g. Fe concentration of whole kernel sample was 15.4 µg/g. P concentration in whole grain sample was 2159 µg/g. * µg/mL. ** µg/g.
